# Fine visualization of biological cells using X-ray micro-CT with the slow freezing contrast improved method

**DOI:** 10.1038/s41598-025-09070-3

**Published:** 2025-07-29

**Authors:** Akio Yoneyama, Masahide Kawamoto, Midori Yasuda, Tatsuhiko Kodama, Kazuhiko Shiranita, Rika Baba, Yuji Matsumoto, Thet-Thet Lwin, Satoshi Takeya

**Affiliations:** 1 Saga Light Source, 8‑7 Yayoigaoka, Tosu, 841‑0005 Japan; 2 Research and Development Group, Hitachi Ltd., 1‑280 Higashi‑Koigakubo, Kokubunji, 185‑8601 Japan; 3 Department of Health and Nutrition Sciences, Nishikyushu Univsersity, 4490-9 Ozaki, Kanzaki, 842-8585 Japan; 4 Saga Prefectural Fruit Tree Experimental Station, 91 Ogimachiharuke, Ogi-shi, 845-0014 Japan; 5 Department of Endoscopy, Respiratory Endoscopy Division/Department of Thoracic Oncology, National Cancer Center Hospital, 5-1-1 Tsukiji, Chuo-ku, Tokyo, 104-0045 Japan; 6 School of Allied Health Sciences, Graduate School of Medical Sciences, Kitasato University, 1-15-1 Kitasato, Minamiku, Sagamihara, Kanagawa 252-0373 Japan; 7 Energy Process Research Institute, National Institute of Advanced Industrial Science and Technology (AIST), Tsukuba West, 16-1, Onogawa, Tsukuba, 305-8569 Japan

**Keywords:** Imaging, 3-D reconstruction, X-ray tomography, Biomedical engineering, Cellular imaging, Imaging techniques

## Abstract

X-ray computed tomography (CT) is widely used in various fields for the non-destructive three-dimensional (3D) observation of internal structures within objects. However, biological cells are primarily composed of light elements such as oxygen and carbon, which have high X-ray transmittance. Consequently, conventional absorption contrast X-ray CT is unable to achieve fine 3D observations of such specimens. We hereby present a technique of novel contrast improvement, the slow freezing contrast improvement method. This method utilizes the aggregation of solutes during slow freezing of aqueous solutions to increase contrast. As plant cells are slowly cooled, intracellular fluid crystallizes, concentrating sugars in specific areas. This process allows for micron-scale visualization of cell structures without staining, using conventional absorption contrast X-ray CT. Experiments on slowly frozen fruits and formalin-fixed mouse organs using synchrotron-based cryo micro-X-ray CT produced high-resolution images of cellular structures. The ice crystal patterns formed within cells varied based on sugar concentration, suggesting potential for detecting sugar levels in individual cells. This method shows promise as a third approach for fine 3D observation of biological cells, complementing contrast agent and phase-contrast imaging techniques.

## Introduction

Biological cells are the fundamental units of organisms, and their structure is crucial information not only in basic medical science but also in a wide range of fields from diagnostics to regenerative medicine. Micro X-ray-computed tomography (CT) has been widely used in various fields—from biomedical to industrial applications—as a non-destructive and three-dimensional (3D) method to observe the inner structures of samples with high spatial resolution in the micron order. However, light elements such as carbon, oxygen, and nitrogen, which are the main constituents of biological soft tissues, absorb few hard X-rays, making fine observation impossible to perform by conventional absorption-contrast X-ray CT, whether laboratory-based or synchrotron radiation-based X-ray CT. To address this limitation, scientists have studied methods utilizing contrast agents^1,2^ such as iodine and osmium, which have greater X-ray absorption. However, contrast agents generally have high viscosity, which limits the areas where contrast can be applied, and in addition the sample is damaged more than usual by X-ray irradiation, which makes performing accurate genetic analysis^3^ difficult. Phase-contrast X-ray imaging that uses X-ray phase shift caused by passing through the sample has also been studied to increase the sensitivity^4–6^. The phase-shift cross-section for light elements is more than approximately 1000× greater than their absorption cross-section, enabling fine observation of biomedical samples and organic materials. However, this method requires highly brilliant and spatially coherent X-rays, such as synchrotron radiation, or an X-ray optical device^7,8^ to bring out fully its high sensitivity, raising the threshold for practical observations.

Freezing meat, fish, and vegetables is known to improve image contrast due to the growth of ice crystals, and fine observations have been performed using conventional CT^9,10^. Furthermore, frozen animal cells and tissues, such as hearts, tendons^11^, and kidneys^12^ reportedly can be observed very clearly with laboratory micro-CT when combined with contrast agents. This is assumed to be due to the formation of ice crystals in the cells during the slow freezing process, which causes various solutes and contrast agents in the intracellular fluid to aggregate, improving the image contrast of CT.

We focused on the fact that plant cells are mostly occupied by vacuoles, and slow freezing can cause ice crystals to grow slowly in the intracellular fluid, causing sucrose and fructose to condense locally, for example, near the cell wall. We expect that the image contrast will be improved, enabling the fine observation of cell structures using conventional CT without any contrast agents. To determine if this contrast-improvement method (slow freezing contrast improvement method) is effective, we carried out experimental observations on small pieces of various slowly frozen fruits using a synchrotron radiation-based cryo-micro–X-ray CT^13^ developed at the Saga Light Source in Japan.

## Results

**Fig. 1:**
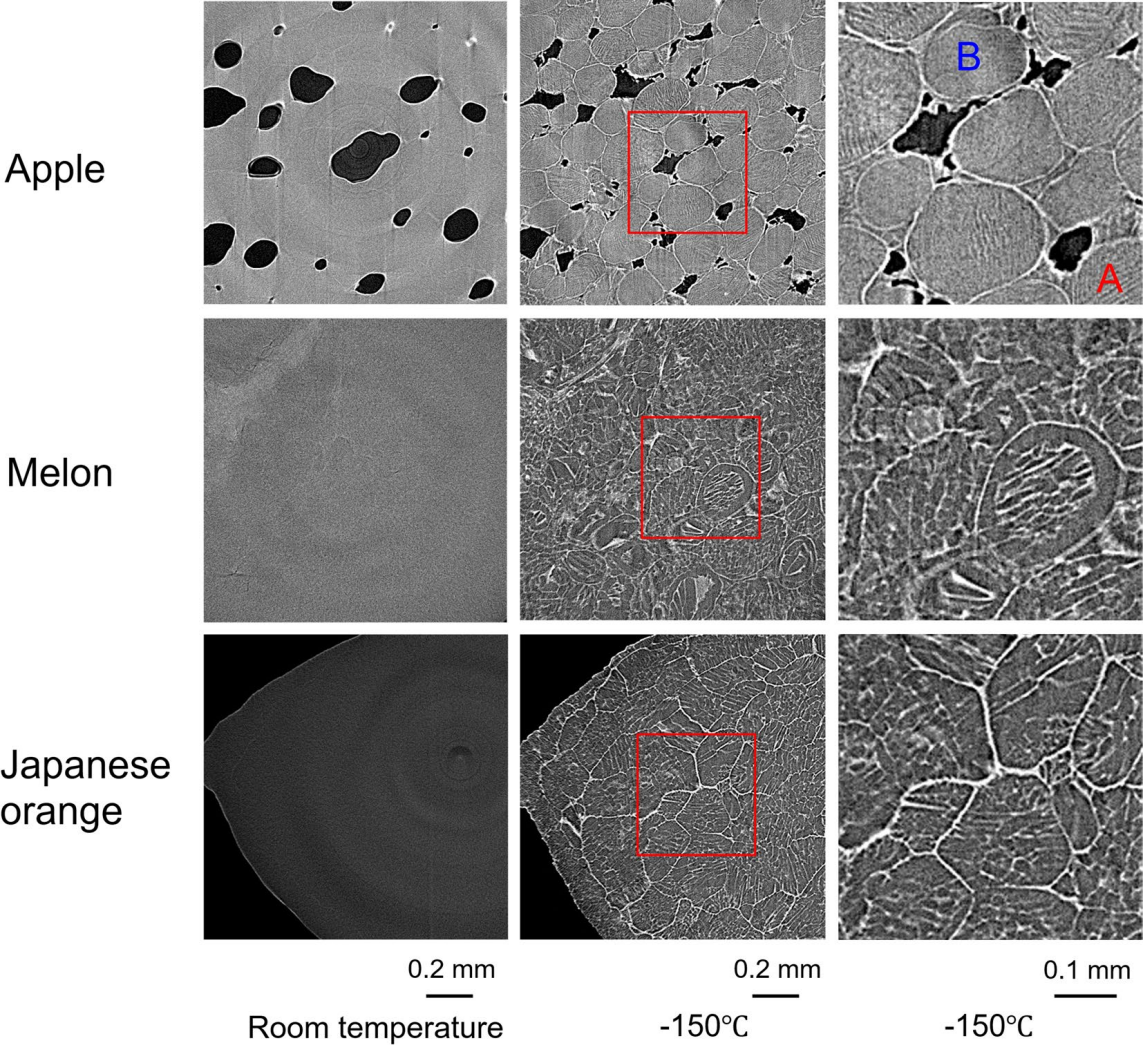
Cross-sectional images of an apple, melon, and mandarin orange at room temperature (left) and slowly frozen at − 150 °C (middle). The magnified image indicated by the red rectangle is shown in right column.

Small pieces of an apple (‘Sun Fuji’), melon (‘Yubari’), and Japanese orange (‘Satsuma Mikan’) (2 mm diameter) were first observed at room temperature and then frozen slowly to − 150 °C and observed again at − 150 °C using a cryo-micro X-ray CT with 10-keV-X-ray-energy described in the method. The left column of Fig. 1 shows the cross-sectional images obtained at room temperature, the middle column shows such images obtained at − 150 °C, and the right column shows an enlarged image of the area indicated by the red rectangle in the middle column images. The results revealed that none of the structures can be visualized in the images obtained at room temperature, whereas various structures are clearly depicted in the slowly frozen (− 40 °C/min) images. Furthermore, the structures are circular in shape and appear to be cells with a stripe-like pattern (region A) and a rough sandy pattern (region B, where the ice crystal pattern is too small to be resolved by the CT used) in the magnified images. White is the high-density area, and black is the low-density area; therefore the small black areas in the apple correspond to air bubbles. Note that the voids in the frozen apple were smaller than those at room temperature, which was likely due to the effect of ice volume expansion.

**Fig. 2:**
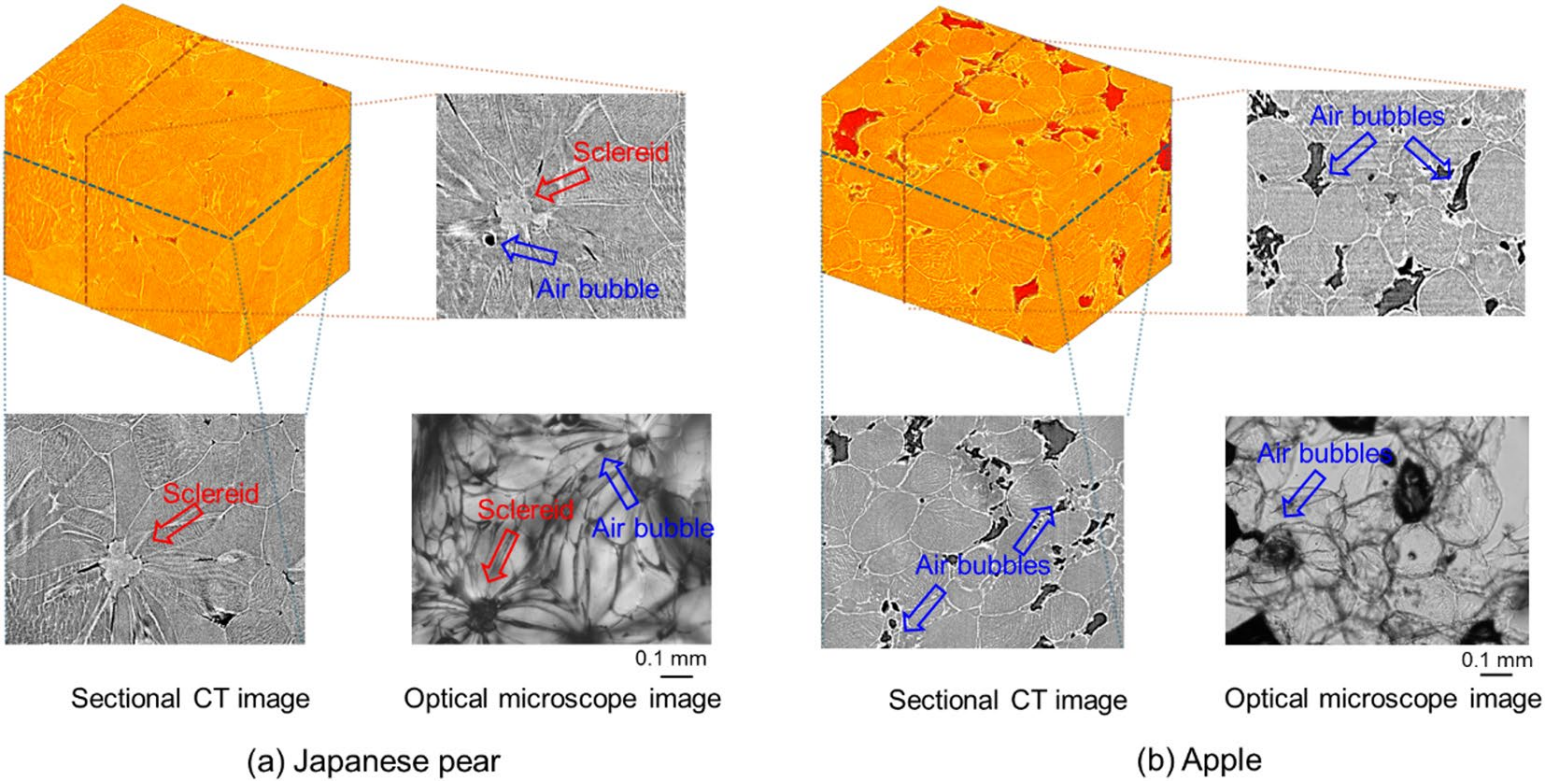
Three-dimensional (3D) volume rendering image (upper left), sectional images in both directions (lower left and upper right) slowly frozen, and optical microscope image (lower right) at room temperature of Japanese pear (**a**) and apple (**b**). 3D images were generated using the Aliza Medical Imaging and DICOM Viewer 2.7.3 at https://www.aliza-dicom-viewer.com/.

Slices of Japanese pears (‘Kosui’) and apples (‘Sun Fuji’), which are relatively rigid even at room temperature, were processed into slices and observed using a visible light (optical) microscope to determine that the circular structures depicted in Fig. 1 were cells. Figure 2 shows the obtained three-dimensional (3D) volume rendering (upper left) and cross-sectional images in the horizontal (lower left) and vertical (upper right) direction of pear (a) and apple (b) slowly frozen, and optical microscopic images (lower right) of sliced pears and apples at room temperature, cut from different parts of the sample before the CT measurement.

The characteristic structure of the sclereids (lithocellular, 100 microns in size) is observable in the Japanese pear microscopic image, and the same shape of the structure is clearly depicted in the CT cross-sectional images of both directions. In addition, the structure of a fan-shaped spread of cells centered on the lithocellular of a similar size (above 0.1 mm in the longitudinally direction) is similarly depicted in microscopic and both CT images. Furthermore, the relatively large size of the round cells (around 0.1 mm) depicted in the apple microscopic image can also be visualized in the CT cross-sectional images in both directions. These observation results led to the conclusion that the circular structures that appear in the cross-sectional images of each of the frozen fruits are the cells of the fruit.

**Fig. 3:**
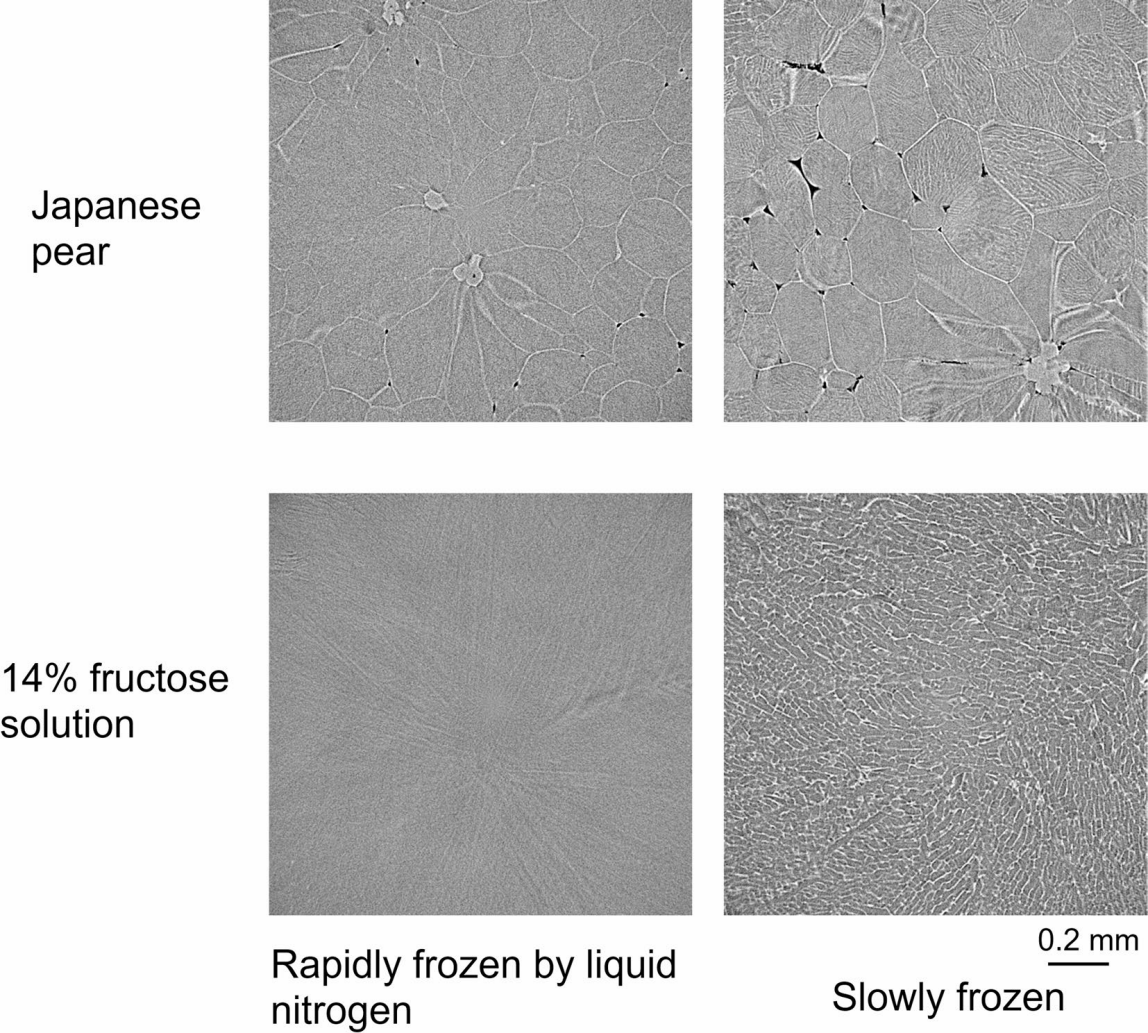
Cross-sectional images of rapidly (left) and slowly (right) frozen Japanese pears (upper) and 14% fructose solution (lower).

The reason for the fine visualization of cells achieved by slow freezing may be that—during the slow freezing process of the liquid in the vacuole (intracellular fluid), which makes up the majority of plant cells—ice crystals grow significantly in the vacuole, and impurities such as fructose and sucrose are deposited and aggregated near the cell wall, resulting in a higher density to improve image contrast. To test this hypothesis, we rapidly froze (< − 150 °C/s) samples of small pieces of Japanese pear (‘Kosui) and of 14% fructose solution by immersing them in liquid nitrogen (LN_2_) and slowly froze (− 40 °C/min) other samples using the cryo system; observations were conducted under the same conditions. The left column of Fig. 3 shows cross-sectional images of the rapidly frozen Japanese pear pieces and fructose solution, and the right column shows those of the same samples slowly frozen.

Support for the aforementioned hypothesis was obtained: the cell wall and sclereids are only slightly visible in rapid frozen Japanese pears, whereas the image contrast of the cell wall was greatly improved and is clearly visualized in slowly frozen Japanese pears. Various patterns (e.g., longitudinal stripes and rough sandy pattern) also appear within the cells. (See the discussion on these patterns.) In addition, the pattern of the rapidly frozen aqueous solution is almost plain, whereas many fine stripes appear in the slowly frozen aqueous solution. These results indicate that the contrast of the cells was improved by an agglomeration of slow freezing.

## Discussion

### Application to the skin-browning symptom of grapes

**Fig. 4:**
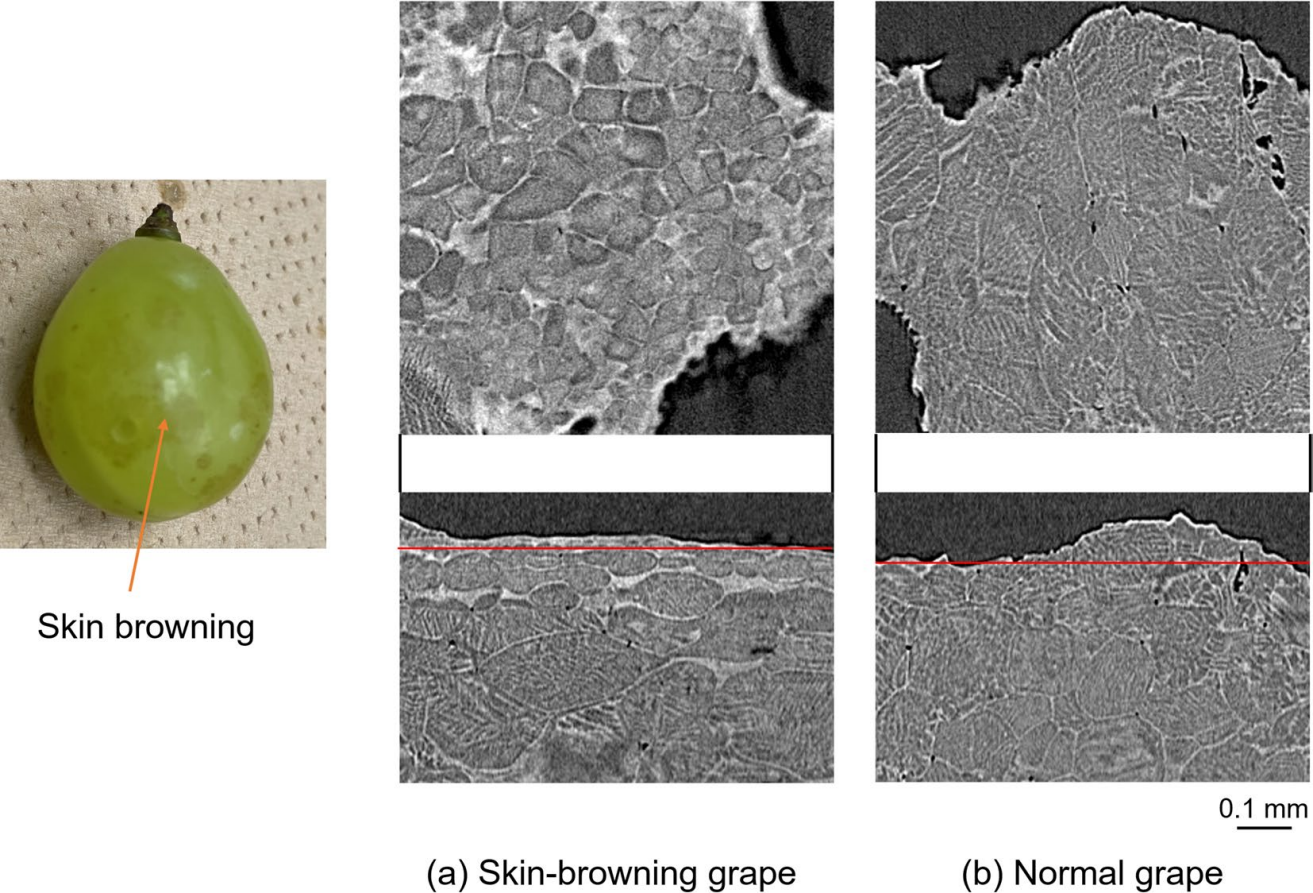
Photograph of a Cheyenne Muscat with the skin-browning symptom (“Kasuri-sho”) (left), (**a**) axial (upper) and sagittal (lower) cross-sectional images of a Kasuri Cheyenne Muscat, and (**b**) the same images of a normal Cheyenne Muscat.

Non-destructive observation of a grape (‘Shine Muscat’ (*Vitis labruscana* Bailey × *V. vinifera* L.)) with a skin-browning symptom (called “Kasuri-sho”) was performed as an application example of the slow freezing contrast improvement method. The skin-browning symptom is a phenomenon in which a part of the grape skin turns brown, as shown on the left of Fig. 4, and it significantly damages the appearance and organoleptic properties of grapes worldwide^14^. Figure 4 shows an axial cross-sectional (upper) and a sagittal cross-sectional (lower) image of a small piece near the surface of a Shine Muscat with Kasuri-sho (a), and the same images of normal Cheyenne Muscat (b). Note that the red line in the sagittal images shows the position of the axial cross-sectional image. The axial image of the Kasuri-sho grape shows that the cells in the skin-browning area were smaller than normal cells (~ 50 μm, half the size of normal cells) and denser with a rough sandy pattern inside the cells. The sagittal image of the Kasuri-sho grape also shows that this degeneration occurred in the first few layers of cells (~ 100 μm) from the surface and that no significant difference occurred inside the normal grape. This information is in good agreement with previous observations of section sliced grapes using an optical microscopy^15,16^. Thus, these results show the method enables non-destructive observation of the cell shape and structure, and it should be a powerful visualization method for clarifying the causes of cultivation problems in various fruits, such as “Kasuri-sho.”

### Elucidation of stripe patterns in cells and its application to a single cell sugar concentrate sensor

As shown in the cross-sectional images in the left column of Fig. 1, the pattern of ice crystals formed inside each cell is very different. For example, in the apple, some cells are filled with a large stripe-like pattern (region A), while others are a rough sandy pattern with no stripe-like structures (region B, where the ice crystal pattern was too small to be resolved by the CT used). These patterns were assumed to be related to the sugar concentration of the intracellular fluid contained in the vacuole of each cell. Specifically, we thought that in intracellular fluid with a high sugar concentration, ice crystal growth was slow due to the water’s low diffusion velocity, resulting in small crystals and the formation of a fine stripe or rough sandy pattern, whereas in intracellular fluid with a low concentration, ice crystal growth was fast and crystal size increased, resulting in the formation of a large stripe pattern. To test this hypothesis experimentally, we slowly froze aqueous solutions of fructose and sucrose at different concentrations under the same conditions, and the stripe patterns were investigated in the cross-sectional images.

**Fig. 5:**
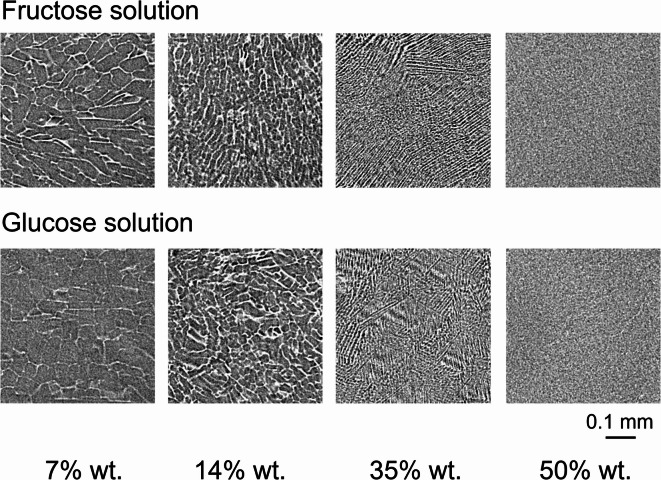
Cross-sectional images of slowly frozen fructose (top row) and glucose solution (bottom row) at different concentrations. The higher the concentration, the finer the stripe spacing, and at 50% the micro- X-ray CT used was unable to resolve the stripes, resulting in a coarse sand-like pattern being observed.

Figure 5 shows the obtained cross-sectional images of fructose (top) and aqueous glucose solutions at different concentrations (7, 14, 35, and 50 wt%). The results show that the higher the concentration, the finer the stripe pattern became, and finally, when the concentration exceeded 50%, the pattern had a sand-like pattern because the micro–X-ray CT used in this study could not resolve such a small pattern. The X-ray diffraction results also showed that the size of the ice crystals formed decreased as the sugar concentration increased^17^, which is qualitatively consistent with these results. The results indicate that the stripe pattern appearing in the cell corresponds to the sugar concentration and that a “single cell sugar content sensor” enabling the detection of the sugar content of each cell is possible by analyzing the stripe pattern of each cell.

**Fig. 6:**
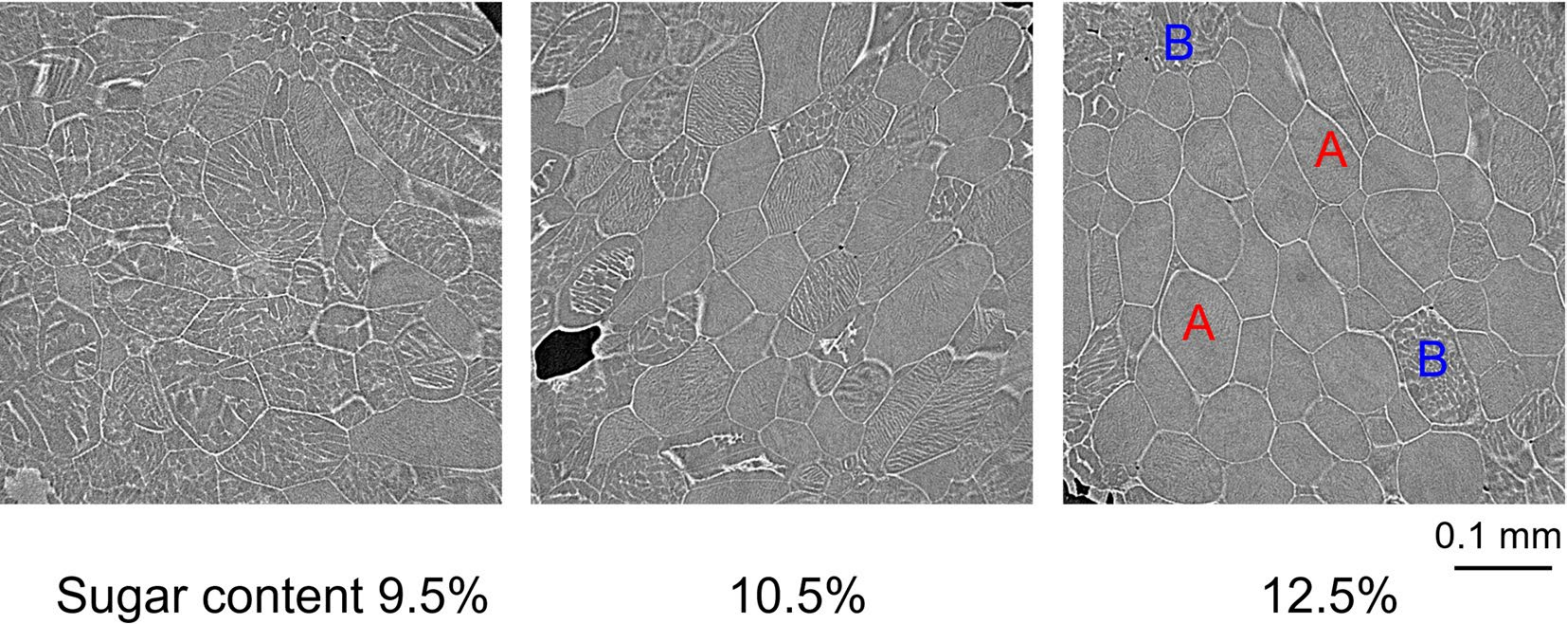
Cross-sectional images of small pieces of Japanese pear with different levels of sugar content.

We conducted a feasibility study of the “single cell sugar content sensor” described, specifically focusing on the relationship between the stripe patterns that appeared in the cells and the sugar content measured by a conventional sugar content sensor. Thus, 12 Japanese pears (‘Kosui’), of different sugar content were studied using the procedure described in the Method section. Figure 6 shows examples of cross-sectional images of small Japanese pear pieces with different levels of sugar content. The ice crystal patterns appearing inside the cells qualitatively had more rough sandy patterns the higher the sugar content. Note that the cell wall is visualized clearly in all images, regardless of the sugar concentration. The cell wall is mainly composed of cellulose, which undergoes only minor changes in density upon freezing, whereas the vacuolar water inside the cell experiences substantial changes in density when frozen. As a result, the density difference between the two materials is relatively significant compared with that at room temperature, and this was thought to lead to fine visualization of the cell wall.

Each cell appearing in the obtained cross-sectional images (every 100 slices (260 microns interval), total 8 images/piece) was manually classified into two patterns: stripe (A) and sandy like (B). Also, the stripe pattern ratio *r*_*a*_ (*r*_*a*_ = B/A) was calculated from the respective cell counts for 12 Japanese pears.

**Fig. 7:**
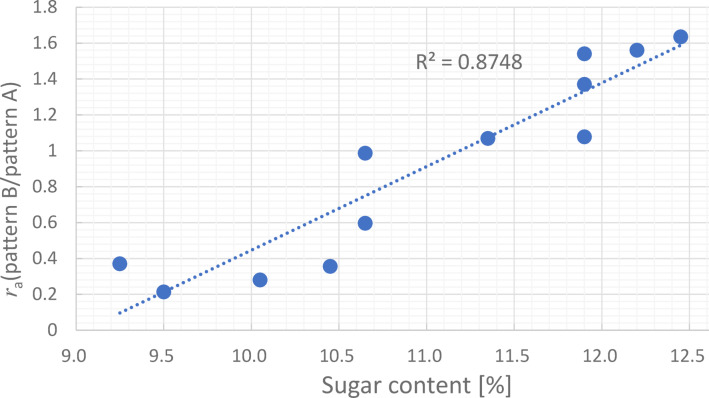
Relationship between sugar content and *r*_*a*_ obtained in 12 Japanese pears with different sugar content.

Figure 7 shows the relationship between the sugar content of the pulp around each small piece and *r*_*a*_. The results show a positive correlation between the sugar content and *r*_*a*_ (the correlation coefficient *R*^2^ = 0.88), suggesting that quantitatively detecting the sugar content of each cell from the stripe pattern of ice crystals is possible in principle. Therefore, a “single cell sugar content sensor” is expected by analyzing and learning the relationship between the stripe pattern and sugar content in detail using numerical methods, such as Fourier analysis and machine learning.

### Feasibility observation for animal cells

**Fig. 8:**
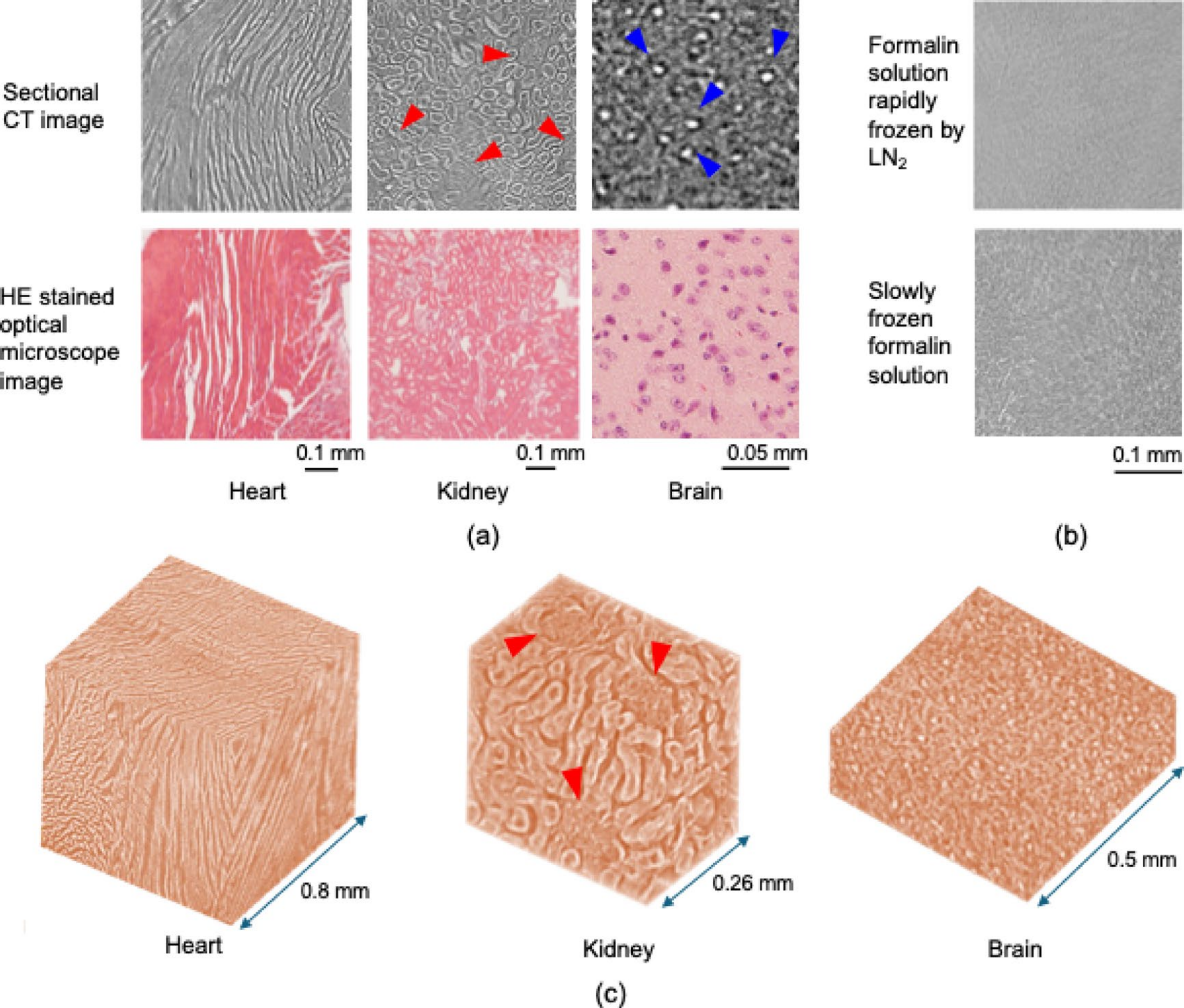
(**a**) Cross-sectional CT images (upper row) and Hematoxylin Eosin (HE)-stained images (lower row) of a mouse heart, kidney with glomeruli (red arrows), and brain with tiny cells (blue arrows) at the same scale. (**b**) Cross-sectional CT images of 4%-formalin solution rapidly frozen by LN_2_ (upper) and slow frozen (lower). (**c**) 3D volume rendering images of heart, kidney, and brain.

Finally, feasibility observations of a heart, kidney, and brain of mice slowly frozen to − 150 °C were performed to test the applicability of this contrast-improvement method with animal cells. Figure 8 shows the obtained cross-sectional images of each tissue without staining by any contrast agents and a Hematoxylin Eosin (HE)-stained image (different sample) at the same scale.

These results show that cell structures that could not be observed at room temperature could be visualized without any contrast agents, i.e., myocardium in the heart, glomeruli (indicated by red arrows), renal tubules, and blood vessels in the kidney, and tiny cells (indicated by blue arrows) in the brain. Figure 8b shows sectional images of formalin solution (4%) rapidly frozen by LN_2_ (upper) and slowly frozen by the cryo system (lower). Similar to Fig. 3, the pattern of the rapidly frozen formalin solution is almost plain, whereas many short stripes appear in the slowly frozen formalin solution. This result indicates that not only sugar but also other solutes are aggregated by slow freezing and thereby improving the image contrast. Thus, the aforementioned improved contrast was thought to be due to the effect of formaldehyde being precipitated and densely aggregated on cell membranes, and each of the cell structures was visualized finely.

Figure 8c shows three-dimensional volume rendering images of the heart, kidney, and brain. The three-dimensional myocardial structure and myocardial fiber orientation in the heart, glomerulus (indicated by red arrows) and surrounding tubular structures in the kidney, and tiny cells (indicated by blue arrows) in the brain are clearly visualized, without staining by any contrast agents, as in the cross-sectional image in Fig. 8a. Note that the lower contrast of the glomeruli in the kidney compared with that of the tubules assumed that the growth of ice crystals was inhibited by the complex capillaries network of the glomeruli, so the aggregation of formaldehyde was lower than that in tubular cells.

Therefore, the use of this contrast-improving technique has been shown to improve contrast even in formalin-fixed animal cells and to visualize cell structures at high resolution without the use of any contrast agent. Although not visualized in Fig. 8 due to the insufficient spatial resolution of the micro X-ray CT used in this study, the intracellular subcellular organelles were assumed to be severely damaged by the growth of ice crystals due to slow freezing. This method would be suitable for non-distracting 3D observation of tissue structures and organs.

## Conclusion and future perspective

In summary, the slow-freezing contrast-improvement method has enabled observing plant cells non-detractively in a contrast-free environment, which had conventionally been difficult to observe. We also found that the pattern of ice crystals in the cells (stripes and rough sandy) was related to the sugar concentration. In the future, quantitatively detecting the internal sugar content of individual cells should be possible using machine learning of the relationship between the pattern and sugar content in CT images. Such advances promise to contribute substantially to the development of fruits with higher sugar content, for example, by studying in detail the relationship between the three-dimensional distribution of sugar content in the fruit, the growing conditions, harvest time, and the position of the fruit on the fruit tree (e.g., the distance from the trunk). In addition, how sugars change in each cell during the ripening and decomposition processes may be revealed, providing guidelines for optimizing storage conditions to reduce food loss.

Furthermore, we found that, even in formalin-fixed animal cells, structures such as renal tubules and glomeruli can be visualized in a non-distracting manner on a micron scale using the slow freezing contrast improvement method. Our method is suitable for observing three-dimensional structures on a relatively large scale in the order of millimeters and is expected to become a new diagnostic method that will complement current pathological diagnosis, focusing mainly on intracellular organelles, by clarifying the relationship between various diseases in which tissue structures, such as tumors, change.

In this study, a monochromatic synchrotron-based micro-X-ray CT with 10-keV-X-ray energy was used as a proof of principle, but the measurement principle is the same as the conventional “absorption contrast” method, which images the absorption of X-rays in the sample. Therefore, although significant differences are evident in X-ray intensity, monochromaticity, and divergence angle, we believe that this improvement method can be effective in laboratory-based micro-CT by simply incorporating a cryo system. The barriers to utilizing this method in universities and hospitals are low, and it has great potential for widespread use.

## Methods

### Cryo-micro–X-ray CT at Saga light source

**Fig. 9:**
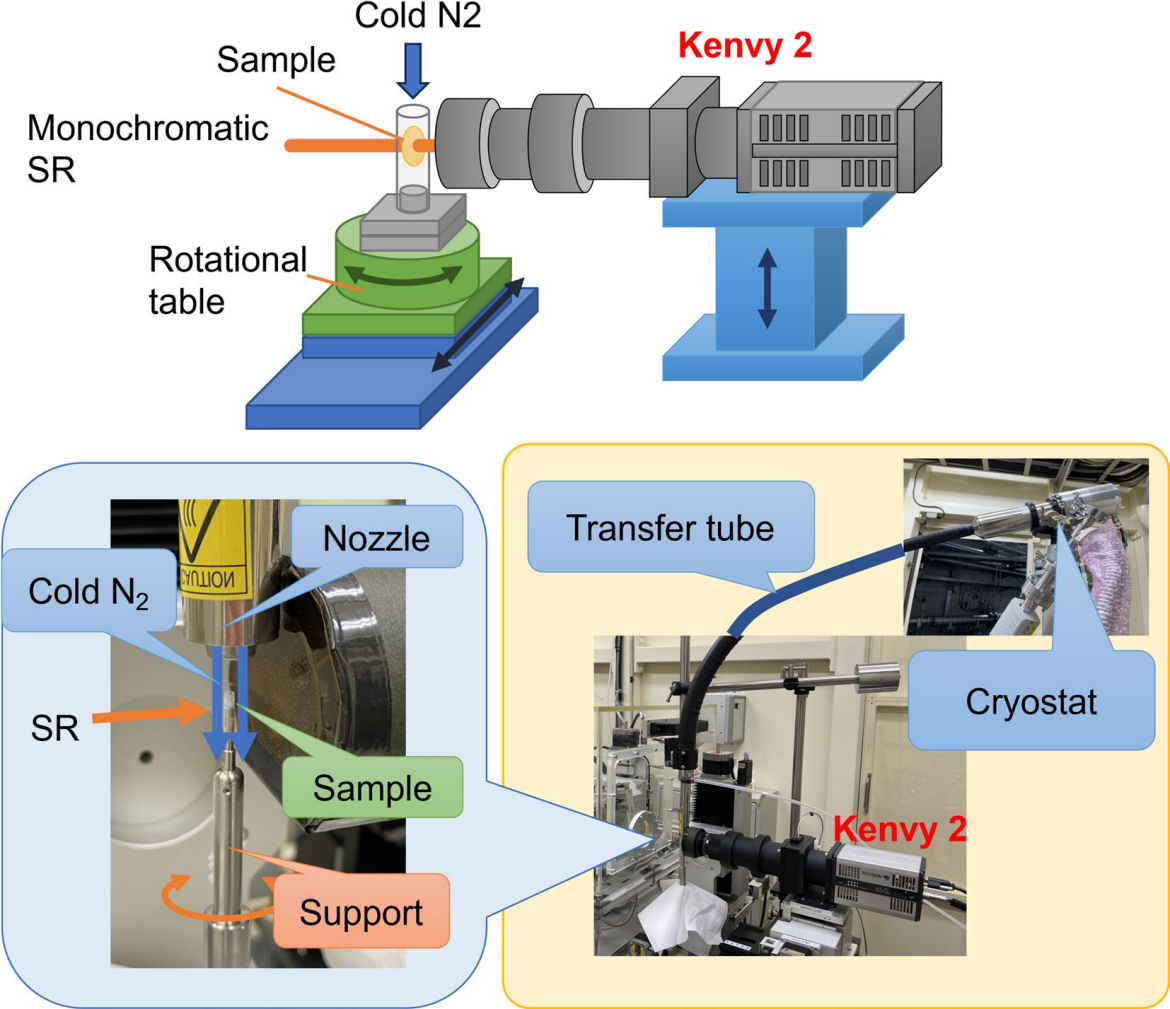
Schematic view and photographs of the cryo micro X-ray CT developed at BL07 of Saga Light Source. High spatial resolution on the order of microns was achieved by shortening the distance between the sample and the X-ray microscopic camera (kenvy2). Figure was generated using the Microsoft PowerPoint 2021 at https://www.microsoft.com/ja-jp/microsoft-365/powerpoint.

Three-dimensional observations were carried out using a cryo-micro–X-ray CT system^13^ (Fig. 9) developed at BL07 of the Saga Light Source (synchrotron radiation facility) in the city of Tosu, Japan. The energy of the monochromatic synchrotron radiation (SR) used was set at 10 keV, the exposure time to obtain a projection image was between 1–2 s, and the number of projections was set between 1000 and 2000 images/360°. The projection images were detected using an X-ray microscopic camera (“kenvy2”) made in house^18^. The camera had a × 5 objective lens, ensuring an effective pixel size of 1.3 μm, 2024 × 2024 pixels, and a field of view of 2.6 mm square. CsI with a thickness of 1 mm was used as the phosphor.

Samples were placed in a 2 mm diameter polypropylene tube to prevent drying during the measurement, and the tube was spun on a rotational table. The samples were cooled by a Rigaku cryo-system (Model GN2) integrated in the aforementioned micro-X-ray CT system, which blew temperature-controlled nitrogen cold gas from the sample top. The temperature of the gas could be set from − 150 to + 80 °C with 0.1 °C temperature stability, and the temperature change rate was 40 °C/min.

First, the sample tube was placed on the rotational table at room temperature (the temperature around the system was controlled at 26 °C), and the CT measurement was performed. The temperature of the nitrogen gas was then slowly reduced from + 20 °C to − 80 °C or − 150 °C at a rate of − 40 °C/min. CT measurements were again performed under temperature-controlled conditions. Each CT cross-sectional image was reconstructed using SAKAS software^19^ with a filtered-back projection method using a Shepp-Logan filter.

In this study, we used 8-week-old Jcl:ICR male mouse, purchased from CLEA Japan Inc. (Tokyo, Japan). Under anesthetized with 2% isoflurane in 1 L/min oxygen delivered through a nose cone, the apex of the left ventricle of the heart was surgically cannulated, and the blood was replaced with physiological saline solution containing heparin to eliminate blood coagulation artifacts within the blood vessels. The brain, kidney, and heart were then rapidly excised and fixed with 4%-paraformaldehyde for cryo-micro CT. The mouse was then euthanized by isoflurane inhalation (5%mg/kg). The animal studies were authorized by the Animal Research and Ethics Committee of Kitasato University (approval number 20-01-3) and were conducted in accordance with the Guidelines for Animal Experiments of Kitasato University and relevant national and international guidelines. All the procedures of the study were followed by the ARRIVE guidelines.

### Measurement of sugar content and calculation of ***r***_***a***_

The sugar content and the pattern ratio *r*_*a*_ appearing in each Japanese pear cell were obtained using the following procedure.

**Fig. 10:**
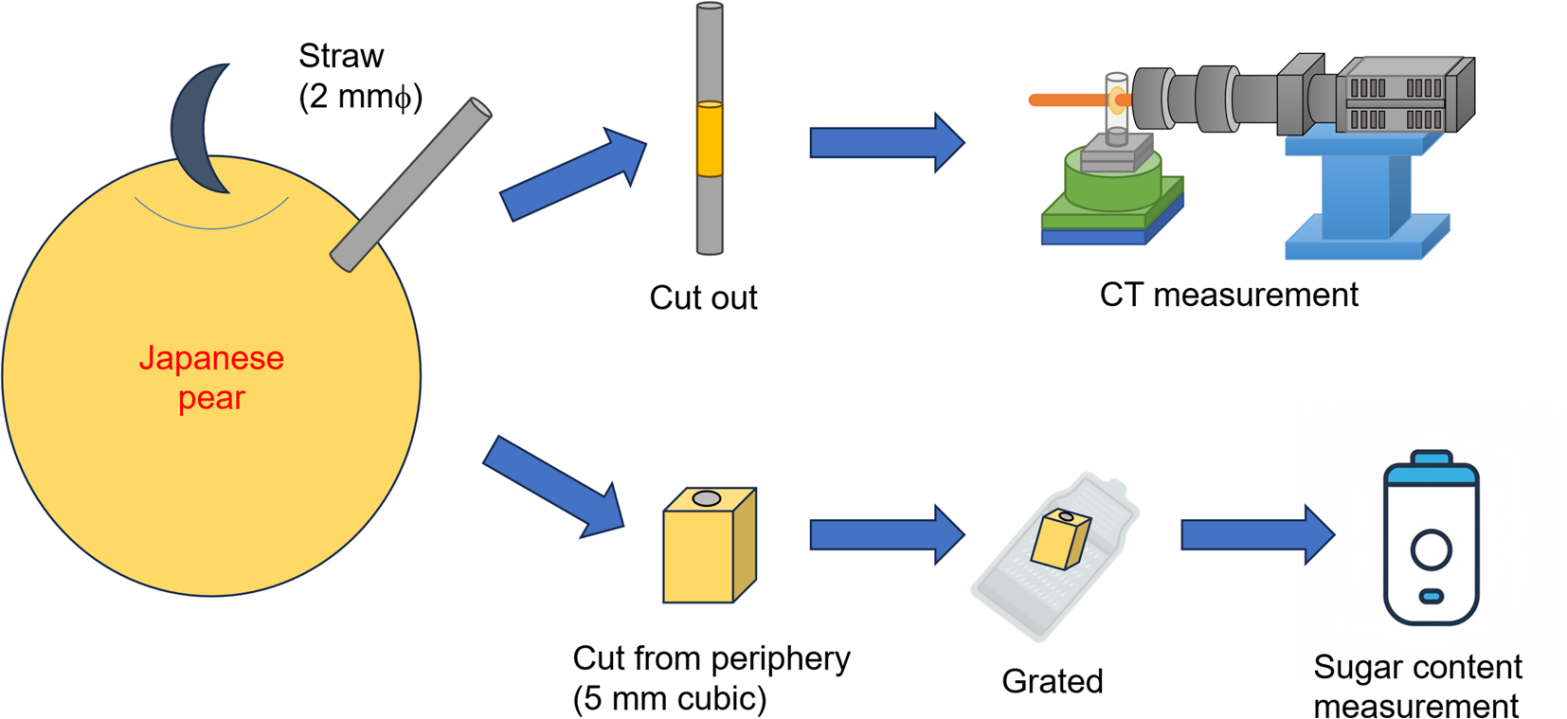
Illustration of procedure (1). A small piece was cut out using a straw for CT measurement, and the surrounding area was grated for a sugar content measurement. Illustration was generated using the Microsoft PowerPoint 2021 at https://www.microsoft.com/ja-jp/microsoft-365/powerpoint.

1. Each Japanese pear (‘Kosui’) was pierced with a straw of 2 mm internal diameter, and a small piece was cut out. Another small piece (5 mm square) cut from the periphery was then grated, and the sugar content of the juice produced was measured using a sugar content sensor (ATAGO Pocket Refractometer PAL-1). (Fig. 10)
2. The straws and small pieces were then cryogenically frozen and observed using micro- X-ray CT at BL07 of Saga Light Source.
3. The ice crystal patterns appearing in the cells of the cross-sectional images were manually classified into one of two types: A: a stripe pattern or B: a sandy pattern. After that, the number of each cell was counted.
4. The stripe pattern ratio *r*_*a*_ (= B/A) was calculated for each pear by performing the procedure (3) for every 100 images (260 microns apart) obtained by (2) to avoid overlapping cells.

## Data Availability

The datasets generated and/or analyzed during the current study are available from the corresponding author upon reasonable request.

## References

[1] Metscher, B. D. MicroCT for developmental biology: A versatile tool for high-contrast 3D imaging at histological resolutions. *Dev. Dyn.* **238**, 632–640. 10.1002/dvdy.21857 (2009). 19235724 10.1002/dvdy.21857

[2] Lusic, H. & Grinstaff, M. W. X-ray-computed tomography contrast agents. *Chem. Rev.* **113**, 1641–1666. 10.1021/cr200358s (2013). 23210836 10.1021/cr200358s PMC3878741

[3] Iacconi, C., Xiarcou, M., Piagneri, V. & Ciofini, E. Impact of iodinated contrast media on X-ray-induced DNA damage: A comprehensive review. *Explor. Cardiol.* **2**, 79–87. 10.37349/ec.2024.00023 (2024).

[4] Fitzgerald, R. Phase-sensitive X-ray imaging. *Phys. Today* **53**, 23 (2000).

[5] Momose, A. X-ray phase imaging reaching clinical uses. *Phys. Med.* **79**, 93–102. 10.1016/j.ejmp.2020.11.003 (2020). 33212423 10.1016/j.ejmp.2020.11.003

[6] Yoneyama, A., Baba, R., Lwin, T. T. & Kawamoto, M. Four-type phase-contrast X-ray imaging at SAGA Light Source. *J. Phys: Conf. Ser.* **2380**, 012117. 10.1088/1742-6596/2380/1/012117 (2022).

[7] Momose, A. Demonstration of phase-contrast X-ray computed tomography using an X-ray interferometer. *Nucl. Instrum. Methods Phys. Res. Sect. A* **352**, 622 (1995).

[8] Momose, A. et al. Demonstration of X-ray talbot interferometry. *Jpn. J. Appl. Phys.* **42**, L866–L868. 10.1143/jjap.42.L866 (2003).

[9] Mousavi, R., Miri, T., Cox, P. W. & Fryer, P. J. Imaging food freezing using X-ray microtomography. *Int. J. Food Sci. Technol.* **42**, 714–727. 10.1111/j.1365-2621.2007.01514.x (2007).

[10] Kobayashi, R. & Suzuki, T. Effect of supercooling accompanying the freezing process on ice crystals and the quality of frozen strawberry tissue. *Int. J. Refrig.* **99**, 94–100. 10.1016/j.ijrefrig.2018.11.045 (2019).

[11] Maes, A. et al. Cryogenic contrast-enhanced microCT enables nondestructive 3D quantitative histopathology of soft biological tissues. *Nat. Commun.* **13**, 6207. 10.1038/s41467-022-34048-4 (2022). 36266273 10.1038/s41467-022-34048-4 PMC9584947

[12] Maes, A. et al. X-ray-based 3D histopathology of the kidney using cryogenic contrast-enhanced MicroCT. *Int. J. Biomed. Imaging* **2024**, 3924036. 10.1155/2024/3924036 (2024). 38634014 10.1155/2024/3924036 PMC11022514

[13] Yoneyama, A. et al. Advanced X-ray imaging at beamline 07 of the SAGA light source. *J. Synchrotron. Radiat.* **28**, 1966–1977. 10.1107/S1600577521009553 (2021). 34738952 10.1107/S1600577521009553 PMC8570222

[14] Gambetta, J. M., Holzapfel, B. P., Stoll, M. & Friedel, M. Sunburn in grapes: A review. *Front. Plant Sci.* **11**, 604691. 10.3389/fpls.2020.604691 (2020). 33488654 10.3389/fpls.2020.604691 PMC7819898

[15] Mochida, K. et al. Relationship between skin-browning symptom (called ‘‘Kasuri-sho”) and mineral nutrient contents in the skin of ‘Shine Muscat’ grapes. *Bull. Shimane Agric. Technol. Center* **41**, 41–50 (2013).

[16] Suehiro, Y., Mochida, K., Itamura, H. & Esumi, T. Skin browning and expression of PPO, STS, and CHS genes in the grape berries of ‘Shine Muscat’. *J. Jpn. Soc. Hortic. Sci.* **83**, 122–132. 10.2503/jjshs1.CH-095 (2014).

[17] Uchida, T. & Takeya, S. Powder X-ray diffraction observations of ice crystals formed from disaccharide solutions. *Phys. Chem. Chem. Phys.* **12**, 15034–15039. 10.1039/c0cp01059f (2010). 20957238 10.1039/c0cp01059f

[18] Yoneyama, A., Baba, R. & Kawamoto, M. Quantitative analysis of the physical properties of CsI, GAGG, LuAG, CWO, YAG, BGO, and GOS scintillators using 10-, 20- and 34-keV monochromated synchrotron radiation. *Opt. Mater. Express* **11**, 398. 10.1364/ome.409161 (2021).

[19] Yoneyama, A. *et al.* in *European Congress of Radiology* (2023).

